# Methamphetamine enhancement of HIV-1 gp120-mediated NLRP3 inflammasome activation and resultant proinflammatory responses in rat microglial cultures

**DOI:** 10.21203/rs.3.rs-3707515/v1

**Published:** 2023-12-16

**Authors:** Debashis Dutta, Jianuo Liu, Enquan Xu, Huangui Xiong

**Affiliations:** University of Nebraska Medical Center; University of Nebraska Medical Center; University of Nebraska Medical Center; University of Nebraska Medical Center

**Keywords:** Methamphetamine, HIV-1gp120, NLRP3 inflammasome, microglia, neuroinflammation

## Abstract

**Background:**

Human Immunodeficiency Virus type 1 (HIV-1)-associated neurocognitive disorders (HAND) remain prevalent in HIV-1-infected individuals despite the evident success of combined antiretroviral therapy (cART). The mechanisms under HAND prevalence in the cART era remain perplexing. Ample evidence indicates that HIV-1 envelope glycoprotein protein 120 (gp120), a potent neurotoxin, plays a pivotal role in the HAND pathogenesis. Methamphetamine (Meth) abuse exacerbates HAND. How Meth exacerbates HAND is not fully understood. This study was to test the hypothesis that Meth exacerbates HAND by enhancing gp120-mediated proinflammatory responses in the brain, worsening the pathogenesis of HAND.

**Methods:**

Experiments were carried out on primary microglial cultures prepared from neonatal SD rats. The purity of microglia was determined by staining with anti-CD11b. Meth and gp120 were applied to microglial cultures. Microglial activation was revealed by immunostaining and Iba-1 expression. The protein expression levels of Pro-IL-1β, Il-1β, Iba-1, iNOS, NLRP3, GSDMD and GSDMD-N were detected by western blotting analyses. The levels of proinflammatory cytokine and NO production in the microglia culture supernatants were assayed by ELISA and Griess reagent systems, respectively. NLRP3 activation was uncovered by fluorescent microscopy images displaying NLRP3 puncta labeled by anti-NLRP3 antibody. NLRP3 co-localization with caspase-1 was labeled with antibodies. One-way ANOVA with post hoc Tukey’s multiple comparison tests was employed for statistical analyses.

**Results:**

Meth enhanced gp120-induced microglia activation revealed by immunostaining and Iba-1 expression, and potentiated gp120-mediated NLRP3 expression, IL-1β processing and release assayed by immunoblot and ELISA. Meth also augmented the co-localization of NLRP3 and caspase-1, increased the numbers of NLRP3 puncta and ROS production, elevated levels of iNOS expression and NO production, and enhanced levels of cleaved gasderminD (GSDMD-N, an executor of pyroptosis) in gp120-primed microglia. The Meth-associated effects were attenuated or blocked by MCC950, an NLRP3 inhibitor, or Mito-TEMPO, a mitochondrial superoxide scavenger, indicating the involvement of mitochondria in Meth enhancement of NLRP3 inflammasome activation in gp120-primed microglia.

**Conclusions:**

These results suggest that Meth enhanced gp120-associated microglial NLRP3 activation and resultant proinflammatory responses via mitochondria-dependent signaling.

## Introduction

The global epidemic of Human Immunodeficiency Virus type 1 (HIV-1) infection and acquired immunodeficiency syndrome (AIDS) remain worldwide public health issues. After more than 40 years of intensive research, we are still far from an HIV-1 cure due to viral reservoirs [1, 2] and no successful vaccine available against HIV-1 despite enormous attempts [3–5]. Although the introduction and widespread availability of combined antiretroviral therapy (cART) have converted HIV-1 disease from a death sentence to a manageable chronic illness [6–10], HIV-1-associated neurocognitive disorders (HAND) remain prevalent in infected individuals. The mechanisms under HAND prevalence in the cART era are not fully understood. A plethora of evidence indicates that the mechanisms are multifactorial, including, but not limited to, virus persistence in the brain, reduced CNS penetration of the cART, viral protein neurotoxicity, chronic neuroinflammation and increased life expectancy [11–14], as well as comorbid factors such as drugs of abuse. Amongst these disease-inciting factors, viral proteins and drugs of abuse play essential roles in HAND pathogenesis and prevalence.

HIV-1 envelope glycoprotein protein 120 (gp120) is a potent neurotoxin that plays a pivotal role in the HAND pathogenesis. Shed off from virions and released from infected cells [13, 15], gp120 accumulates in the cerebrospinal fluid and brain tissue in a significant amount and causes neuronal damage both in vitro and in vivo [16, 17]. Studies have shown that gp120, on the one hand, induces neuronal apoptosis, synaptic and dendrite dysfunction [18, 19] and, on the other hand, triggers immune activation and resultant production of neurotoxic molecules as well as inflammasome-dependent pyroptosis [20], leading to the development of HAND [15, 21, 22]. The neurocognitive impairment observed in patients with HAND can be attributed to direct and indirect neurotoxic effects of gp120 and are frequently associated with and worsened by abuse of recreational drugs such as methamphetamine (Meth) [23, 24].

Meth is a potent and highly addictive psychostimulant that is frequently used by HIV-1-infected individuals [25]. Meth abuse not only increases the risk of HIV-1 transmission but also augments HIV-1-associated neurocognitive impairments. [26–28]. Ample evidence indicates that Meth exacerbates HAND [23, 29, 30]. While many studies have focused on their individual effects on the CNS, much less have studied their comorbid influence on HAND pathogenesis. We have previously reported that Meth potentiated gp120 enhancement of microglial outward K^+^ current, leading to increased production of proinflammatory molecules and consequent neuronal injury [31]. We also showed in another study that microglial nucleotide-binding domain, leucine-rich–containing family, pyrin domain–containing-3 (NLRP3) inflammasome was involved in Meth potentiation of gp120 inhibition of long-term potentiation, a widely accepted synaptic mechanism for learning and memory, implying a potential mechanism for Meth exacerbation of HAND seen clinically [32].

Inflammasomes are cytosolic multiprotein signaling complexes that trigger the activation of inflammatory caspases and the maturation of IL-1β. They are critical for the host’s immune defense against microbial infection and cell injury. Among various inflammasome complexes, the NLRP3 is an extensively studied and well-characterized inflammasome [33, 34], which recognizes various stimuli via NOD like receptor (NLR) and serves as a platform for caspase-1 activation. Activation of the NLRP3 inflammasome requires two signals: the first signal (priming signal) activates the transcription factor NF-κB leading to upregulation of NLRP3 and pro-IL-1β. The second signal (activation signal) consists of a variety of stimuli that promote the assembly of ASC and procaspase-1 and results in activation of the NLRP3 inflammasome and caspase-1 [35–38]. The activated caspase-1 cleaves pro-IL-1β, pro-IL-18 and gasderminD (GSDMD), resulting in the release of matured IL-1β, IL-18 and GSDMD-N (a N-terminal fragment of GSDMD) and consequent inflammation and pyroptosis [34, 36, 39]. It is our hypothesis that Meth exacerbates HAND via potentiation of HIV-1-associated microglial inflammasome activation and resultant proinflammatory responses. To test this hypothesis, we investigated the effects of Meth on NLRP3 inflammasome activation and resultant proinflammatory cytokine production in gp120-primed rat microglial cultures. Our results showed that Meth enhanced gp120-stimulated microglial activation and resultant cytokine production via mitochondria-dependent NLRP3 inflammasome activation.

## Material and Methods

### Materials

Full-length HIV-1 envelope glycoprotein 120 from a Clade B virus (HIV-1MN gp120) was purchased from Immunodiagnostics, Inc. (Woburn, MA) and stored at − 80°C freezer in 100 nM aliquots. Methamphetamine was purchased from Sigma-Aldrich (St. Louis, MO, Cat # M-8750) with DEA license # RX0374974. Lipopolysaccharide (LPS, from *Escherichia coli* 0111:B4) was also procured from Sigma-Aldrich. MCC950 and Mito-TEMPO were obtained from Enzo Life Sciences (Farmingdale, NY). All other chemicals, unless otherwise specified, were purchased from Sigma-Aldrich.

### Animals

For the isolation of microglia from neonates, pregnant female Sprague-Dawley (SD) rats were purchased from Charles River Laboratories (Wilmington, MA). Animals were kept in the University animal house at constant temperature (22°C) and relative humidity (50%) under a regular light-dark cycle (light on at 7:00 AM and off at 5:00 PM) with adequate access to food and water round the clock. All the animal use procedures in the study were strictly reviewed by the Institutional Animal Care and Use Committee (IACUC) of the University of Nebraska Medical Center (IACUC No. 19-085-07-FC).

### Isolation and culture of microglial cells

Microglial cells were isolated from the cerebral cortex of postnatal (0–1 day old) SD rats as described previously [40]. Briefly, rat cortical tissues were dissected out in cold Hank’s Balanced Salt Solution (HBSS: Mediatech, Inc. Manassas, VA) and digested with 0.25% trypsin and 200 Kunitz units/ ml DNase (Sigma-Aldrich) in 37°C for 30 min. The digested tissues were then suspended in cold HBSS and filtered through 100 μM and 40 μm pore cellular strainers (BD Bioscience, Durham, NC), respectively. The isolated cells (25 × 10^6^) were plated into T75 cm^2^ flasks in a high-glucose Dulbecco’s modified Eagle’s medium (DMEM) containing 10% fetal bovine serum (FBS), 1×glutaMAX, 1% penicillin/streptomycin (LifeTechnologies, Grand Island, NY), and 300 ng/ml macrophage colony-stimulating factor (M-CSF) supplied by the Department of Pharmacology and Experimental Neuroscience, University of Nebraska Medical Center. After 8–10 days in culture, the flasks were gently shaken and the detached cells were collected and seeded onto 6 well (2 × 10^6^ /well) and 12 well (1 × 10^6^ /well) or 96 well plates (0.5 × 10^6^/well) based on the experimental requirements with M-CSF free DMEM. The suspensory glial cells were removed 1 h after seeding by changing the culture media. The resultant cultures were 98–100% microglia as determined by staining with anti-CD11b (Abcam, Cambridge, MA), a marker for microglia.

### Western Blotting

After priming with gp120 for 48 h, microglial cells were treated with Meth for another 24 h. The cells were then lysed using RIPA buffer (Bio-Rad, Hercules, CA) for analyzing pro-IL-1β processing. 30 μg total proteins were separated by 4–20% gradient PAGE and transferred to nitrocellulose polyvinylidene difluoride (PVDF) membranes. The membranes were blocked with 3% bovine serum albumin (BSA) in tris-buffered saline (TBS) and incubated overnight at 4°C with either rabbit polyclonal antibody to IL-1β at a 1:500 dilution (Abcam, Boston, MA), mouse polyclonal antibody to NLRP3 at a 1:1000 dilution (Adipogen, San Diego, CA), rabbit Iba-1 antibody at 1:1000 dilution (Fujifilm, Richmond, VA) or anti-mouse β-actin monoclonal antibody (1:5000, Sigma-Aldrich). The washing buffer was TBS with 0.2% Tween (TBS-T). The secondary antibody was horseradish peroxidase (HRP)-conjugated anti-rabbit or anti-mouse antibody (1:10000, Jackson Immuno Research Laboratories, PA). Labeled proteins were shown by the Pierce-enhanced chemiluminescence (ECL) system (Thermo Fisher Scientific, Waltham, MA).

### Enzyme-linked immunosorbent assay (ELISA) analysis

Secretion of IL-1β and other cytokines in the culture supernatants was assayed by ELISA. Microglia were primed with gp120 After 48 h priming with gp120 and then stimulated with Meth at different concentrations for another 24 h. The cells were washed three times with PBS before the addition of Meth. After the 48 h priming with gp120, microglia were washed three times before Meth treatment. To detect IL-1β release, the supernatant of Meth-treated microglia was collected at 24 h. Cytokines in supernatants were detected using the ELISA kit (R&D system, MN). The experiments were performed following the manufacturer’s instructions. Briefly, the plates were coated with capture antibody overnight at room temperature and then the reagent dilution buffer was used as the blocking reagent. The capture antibody-coated 96-well plates were incubated with collected supernatants for 2 h at room temperature, followed by 2 h application of the detection antibody. Finally, the Streptavidin-HRP working solution was incubated for 20 min before substrate solution was added to each well. After this reaction was stopped using stop solution and reading was done using a Bio-Rad microplate reader with filters at 450/560nm and the result was calculated using a 4-parametric curve.

### Measurement of nitric oxide (NO) production

The production of nitrite was measured by the Griess reagent system according to the manufacturer’s instructions (Promega, Madison, WI). After treatment of gp120 and Meth, 50 μl aliquots of culture supernatant were collected from each treatment condition. 50 μl of sulfanilamide solution and 50 μl NED solution were added with collected supernatants for 10 min and 10 min separately. The absorbance of the final samples was measured on a Bio-Rad microplate reader with filters at 560 nm.

### Fluorescent dye loading and imaging

Fluorescent probes against ROS production H2DCFDA were loaded onto the microglia that were treated with Meth for an additional 24 h after priming with gp120 for 48 h.

5-(and-6)-chloromethyl-2′,7′-dichlorodihydrofluorescein diacetate (CM-H2DCFDA) (Life Technologies, Eugene, OR) was deployed to examine the intracellular ROS production. CM H2DCFDA enters cells passively and the acetate groups of the probe were cleaved by intracellular esterase, leading to better cellular retention. After oxidation by reactive oxygen intermediates generated in response to Meth, the non-fluorescent H2DCFDA is converted to the highly fluorescent 2′,7′-dichlorofluorescein (DCF). The CM-H2DCFDA (5 μM) working solution was freshly made with a pre-warmed DMEM medium and incubated on treated microglia at 37°C for 30 min. The cells were then fixed with ice-cold 4% paraformaldehyde in PBS for 10 min and counterstained with 4′,6-diamidino-2-phenylindole (DAPI). To quantify the results, the microglia were seeded onto a 96-well black plate at a density of 0.25 × 10^6^/well and the treatment procedure mentioned above was then repeated. After loading with CM-H2DCFDA, the intensities of fluorescent signals were evaluated by the microplate reader.

### Immunocytochemistry

Immunocytochemistry was performed to quantify NLRP3 puncta as a readout of inflammasome activation. Microglia were seeded on coverslips in a 24-well plate at a density of 0.5 × 10^6^ cells per well. After priming with gp120 (0.5 nM), for 48 h Meth treatment was for 24 hrs. Iba-1 staining was conducted to monitor microglial activation. The cells were fixed with 4% paraformaldehyde (PFA) for 10 min at room temperature. Then, the cells were blocked and permeabilized in PBS with 10% goat serum and 0.1% Triton X-100 for 15 min. The primary antibodies used included mouse polyclonal antibody to NLRP3 (Santa Cruz, CA) and rabbit Iba-1(Sana Cruz, Dallas, TX) at 1:100 dilution. The microglia were identified by mouse monoclonal antibody CD11b in 1:500 dilution (Abcam, Cambridge, MA). The secondary antibodies used here were goat anti-rabbit Alexa 488 (1:1000) and Alexa594 (1:1000) from ThermoFisher Scientific (Waltham, MA).

### Data analyses

All data are expressed as mean ± S.D. unless otherwise indicated. Statistical analyses were performed by one-way ANOVA followed by post hoc Tukey’s multiple comparisons test (GraphicPad Prism, version 9.4.1). A minimum *p*-value of 0.05 was chosen as the significance level for all tests. The densities of target western blot bands were quantified using NIH Image J software and standardized by β-actin band density. Percentage of microglia with NLRP3 puncta was estimated by microscopic scoring[41]. In the fluorescent staining for total ROS production, 9 fields under the fluorescent microscope were taken, and all cellular fluorescent intensities were quantified by Image J. The intensities of all individual cells in each field were averaged and transformed to fold changes against a control group. All experiments were performed in triplicate unless otherwise specified.

## Results

### 1. Meth potentiated HIV-1 gp120 - induced IL-1β processing and release

**Figure 1:**
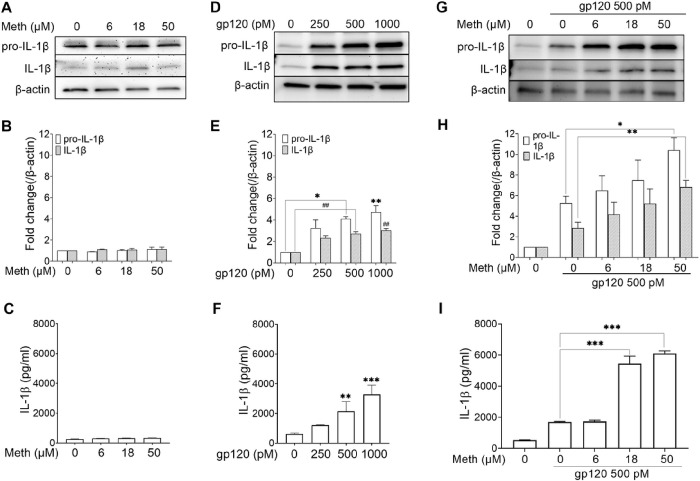
Meth potentiation of HIV-1 gp120-induced IL-1βprocessing and release. Data illustrated in three columns were obtained from rat microglial cultures treated with Meth (left) and gp120 (central), each alone at different concentrations, respectively, or with one concentration of gp120 (500 pM) and different concentrations of Meth (right). The upper row displayed western blot results of the expression levels of pro-IL-1β and IL-1β when microglia were treated with Meth (A) and gp120 (D) alone or gp120 500 pM with different concentrations of Meth (G). Bar graphs in the middle row were corresponding densitometry quantitation of pro-IL-1β, IL-1β on western blots shown in the upper row. The lower row showed the levels of IL-1β detected by ELISA from culture supernatants of microglia under different experimental conditions as indicated. *p<0.05, **p<0.01, ***p<0.001 vs. untreated controls (E, F) or vs. gp120 alone controls (H, I). Data represent mean ± SD derived from three independent experiments in triplicate.

IL-1β processing and release are stringently regulated by inflammasomes [42]. Inflammasome activation occurs in two steps, priming (first signal, transcription and expression of inflammasome components) and processing (second signal, assembly of inflammasome components) [42, 43]. Although HIV-1 gp120 is known to signal both steps [20], an additional second signal can further enhance the activation of gp120-primed inflammasomes. We have previously demonstrated that treatment of microglia with Meth alone had no significant effects on the IL-1β transcript, suggesting Meth does not work as the first signal for inflammasome activation [44]. Consistent with our previous results, treatment of microglia with Meth alone with different concentrations (6, 18, 50 μM) failed to produce significant effects on IL-1β processing detected by immunoblotting (Fig. 1A and B) and IL-1β production measured by ELISA (Fig. 1C). To test the effects of gp120 on inflammasome activation, microglial cells were treated with gp120 at various concentrations (250, 500 and 1000 pM) and concentration-dependent responses were observed. As evident from the western blot results, a significant IL-1β processing occurred at 500 pM (2.7-fold, p < 0.01) and 1000 pM (3-fold, p < 0.01) compared to untreated controls (Figs. 1D, 1E). The IL-1β processing results were parallel with the results of IL-1β release in culture supernatant measured by ELISA (Fig. 1F), illustrating a concentration-dependent IL-1β release mediated by gp120. As 500 pM gp120 increased IL-1β processing and release and 50 μM Meth had no significant effect, we examined if Meth could potentiate gp120 effects on IL-1β processing and release when applied in combination. The results showed that Meth (50 μM) enhanced the IL-1β processing (2.6-fold, p < 0.01) and release (3.7-fold, p < 0.001) in gp120-primed microglia compared to the results from solely gp120-primed microglia (Figs. 1G, 1H and 1I). These results provide us with the optimum concentrations of Meth (50 μM) and gp120 (500 pM) for testing Meth potentiation of gp120 effects on microglial inflammasome activation in this study.

As evident from the western blot results, significant IL-1β processing occurred at 500 pM (2.7-fold, p < 0.01) and 1000 pM (3-fold, p < 0.01) compared to untreated control (Figs. 1D, 1E). IL-1β processing result was further supported by IL-1β release in culture supernatant measured by ELISA (Fig. 1F). A significant dose-response of IL-1β release was observed at 500 pM (3.4-fold, p < 0.01) and at 1000 pM (5.2-fold, p < 0.001) compared to the untreated control sample. As 500 pM gp120 was the “lowest” concentration to produce significant effects on IL-1β processing and release, this concentration was adapted in the experiments exploring Meth potentiation of gp120-mediated microglial proinflammatory responses.

### 2. Meth augments gp120-primed microglia activation

**Figure 2:**
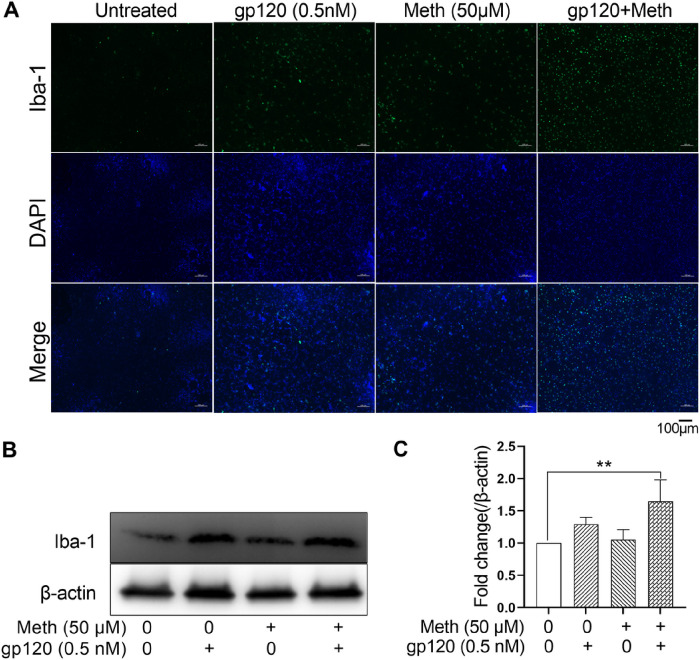
Meth augments gp120-primed microglia activation. (A) immunofluorescence labeling of Iba-1 expression in microglia under different experimental conditions as indicated. Note that an enhanced Iba-1 expression was detected in microglia treated with gp120+Meth. (B) Western blot detection of Iba-1 protein expression and an increased level of Iba-1 expression was observed with the treatment of gp120+Meth. (C) Image J densitometry analyses of Iba-1 protein expression in western blots. **p<0.01 One-way ANOVA followed by Dunnett’s comparisons test. Scale bar equals 100μM.

Ionized calcium-binding adaptor molecule 1 (Iba-1) is a microglia-specific marker and its expression is known to increase upon microglia activation [45, 46]. Immunofluorescence microscopy and immunoblotting revealed that unstimulated rat microglia expressed a low level of Iba-1. In gp120-treated microglia, a remarkable increase in Iba-1 staining was observed (Fig. 2A). The Iba-1 staining was further enhanced after the treatment of gp120-primed microglial cells with Meth (Fig. 2A). In an agreement with immunofluorescence staining, western blot results of cell lysate showed an increased level of Iba-1 in microglia treated with gp120, which was augmented by Meth (Figs. 2B, 2C). Statistical analyses revealed that the levels of Iba-1 expression were significantly (p < 0.01) enhanced when microglial cells were treated with gp120 and Meth in combination compared to those treated with gp120 alone or untreated control. These results demonstrated Meth augmentation of gp120-induced microglia activation.

### The effect of Meth on NLRP3 co-localization and puncta formation

**Figure 3:**
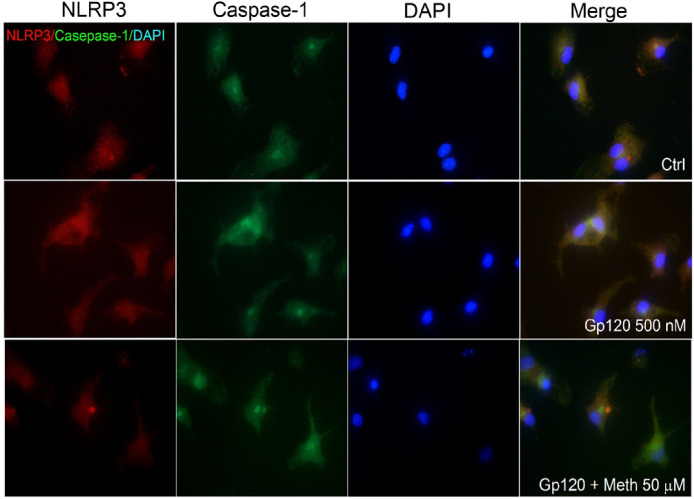
Meth-induced co-localization of NLRP3 and caspase-1 in gp120-primmed microglia. Immunofluorescence images were taken from microglial cultures treated with gp120 (middle row) and gp120+Meth (bottom row) after staining separately with primary antibodies against NLRP3 (red) and caspase-1 (green). The upper row was untreated controls. Note that Meth induced co-localization of NLRP3 and caspase-1 as shown in merged images. Blue in color was a DAPI stain. Images were taken at 60 magnification.

**Figure 4:**
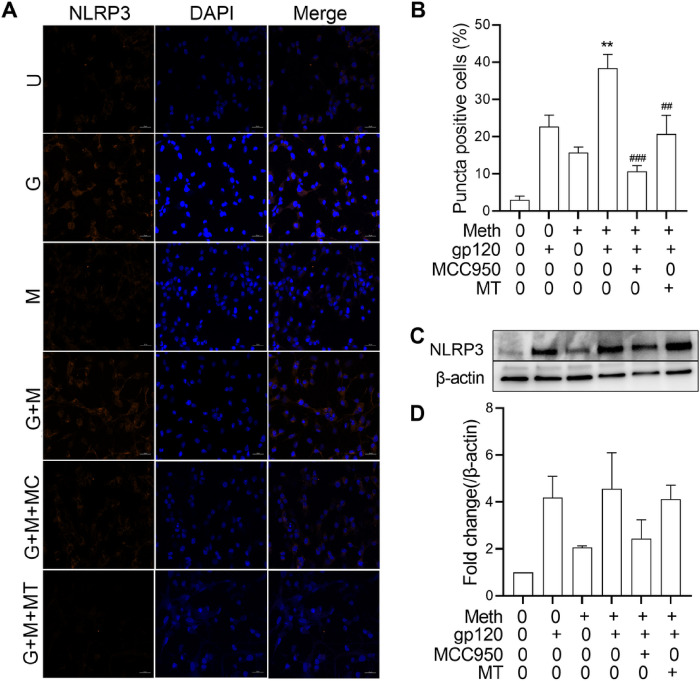
Meth triggers NLRP3 activation in gp120-primed microglia. Panel A: Representative fluorescence microscopy images display NLRP3 puncta (red) labeled by anti-NLRP3 antibody (left column) in different experimental conditions as indicated. Cell nuclei were stained with DAPI (blue, middle column). The right column showed merged images. Panel B: Percentage of microglia containing NLRP3 puncta as estimated by microscopic scoring. Panels C and D: Western blot detection of NLRP3 and corresponding densitometric analysis of NLRP3 from different treatment conditions. Note the synergic effects of Meth and gp120 on the increase of NLRP3 puncta positive cells and NLRP3 expression levels and their significant blockade by MCC 950, an NLRP3 inflammasome activation blocker, or by Mito-TEMPO, a mitochondria-targeted antioxidant. ***p<0.001, ^###^p<0.001, **p<0.01 One-way ANOVA followed by Tukey’s multiple comparisons tests. Abbreviations used in figure: U (untreated), G (gp120), M (Meth), MC (MCC950), MT (Mito-TEMPO).

The NLRP3 inflammasome plays an important role in microglia activation [34]. To evaluate the effect of Meth on NLRP3 inflammasome activation, we examined the co-localization of NLRP3 and caspase-1 in gp120-primmed microglia. Our results showed an increased NLRP3/caspase-1 co-localization upon Meth treatment (Fig. 3), indicating Meth enhancement of microglia NLRP3 inflammasome activation. Meth enhancement of microglia NLRP3 inflammasome activation was further validated by NLRP3 puncta formation visualized by immunofluorescence. We observed a significant increase in NLRP3 puncta formation upon Meth treatment in gp120-primed microglia (Figs. 4A, 4B). Pretreatment of microglial cultures with MCC950 (a selective inhibitor of NLRP3 inflammasome) or Mito-TEMPO (a mitochondria ROS inhibitor) attenuated Meth increase in NLRP3 puncta formation (Figs. 4A, 4B), indicating NLRP3 activation and involvement of induction of ROS upstream signaling. Additionally, Meth was also found to augment gp120-primed increase of NLRP3 expression (Figs. 4C, 4D). Pretreatment of microglial cultures with MCC950 significantly blocked Meth/gp120-associated increase of NLRP3 expression. However, partial blockade of Meth/gp120-associated increase on NLRP3 expression was observed when microglial cultures were pretreated with Mito-TEMPO.

### Meth potentiation of proinflammatory cytokine production in gp120-primed microglia

**Figure 5:**
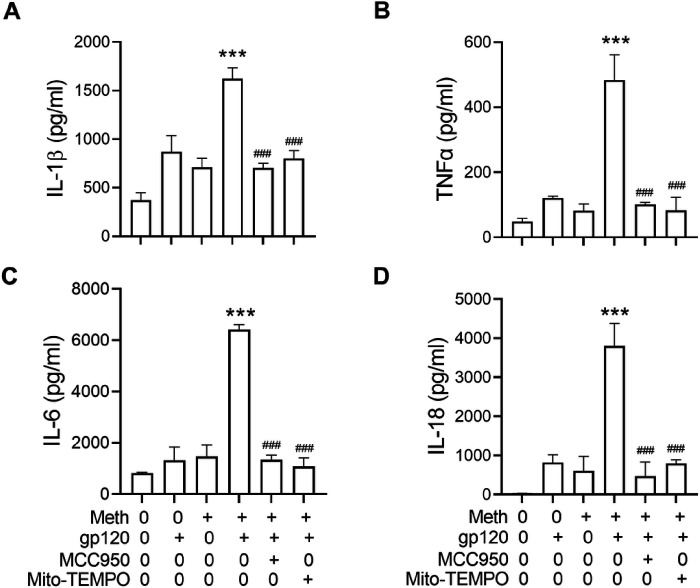
Meth increased proinflammatory cytokine production in gp120-primmed microglia. ELISA analyses of the levels of proinflammatory cytokines in the culture supernatants recovered from microglia with experimental treatments as indicated. Increased production levels of IL-1β (A), TNF-α(B), IL-6 (C) and IL-18 (D) were detected gp120-primed, Meth-treated microglia. Pretreatment of microglia with NLRP3 inhibitor MCC950 or mitochondrial superoxide scavenger Mito-TEMPO significantly decreased the levels of cytokine production. ***p<0.001, ^###^p<0.001.

**Figure 6:**
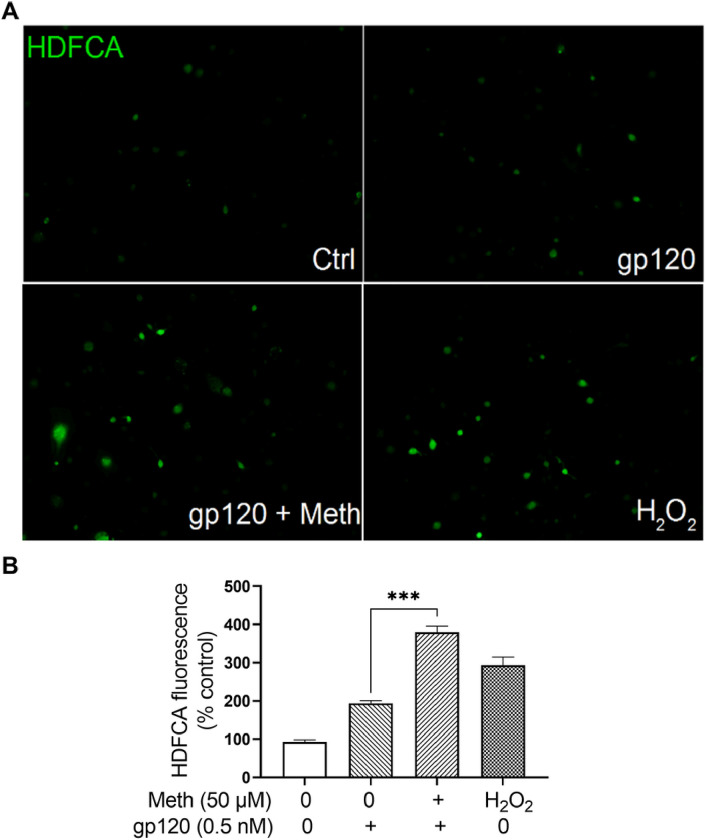
Meth and HIV-1 gp120 synergistically magnify total ROS production. Panel A: Combined stimulation of Meth and gp120 synergistically activated total ROS production. Panel B: Quantified results were displayed in a bar graph. All images were captured at 40X original magnification. ***p<0.001.

It is well established that up-regulation of pro-inflammatory cytokines plays multiple roles in both neurodegeneration and neuroprotection. To examine if Meth potentiation of gp120-primmed microglial NLRP3 inflammasome activation could result in an increase of cytokine production, we detected the levels of TNF-α, IL-1β, IL-6 and IL-18 in the culture supernatants by ELISA. Significant increases in IL-1β (~ 4.35 fold), TNF-α (~ 10 fold), IL-6 (~ 7.81 fold) and IL-18 (~ 55 fold) in gp120-primed and Meth-treated microglia (Figs. 5A, 5B, 5C and 5D) compared to those detected in the supernatants collected from gp120 primed microglia, suggesting Meth potentiation of proinflammatory cytokine release responsible for the neuronal injury [47]. In addition, Meth was found to enhance mitochondrial total ROS production (Figs. 6A, 6B). The Meth-associated increase of cytokine production was blocked by pretreatment of microglial cells with MCC950 or Mito-TEMPO, suggesting involvement of NLRP3 inflammasome and its upstream mitochondrial ROS signaling in Meth-associated potentiation of cytokine release.

### Meth enhancement of iNOS expression and NO production

**Figure 7:**
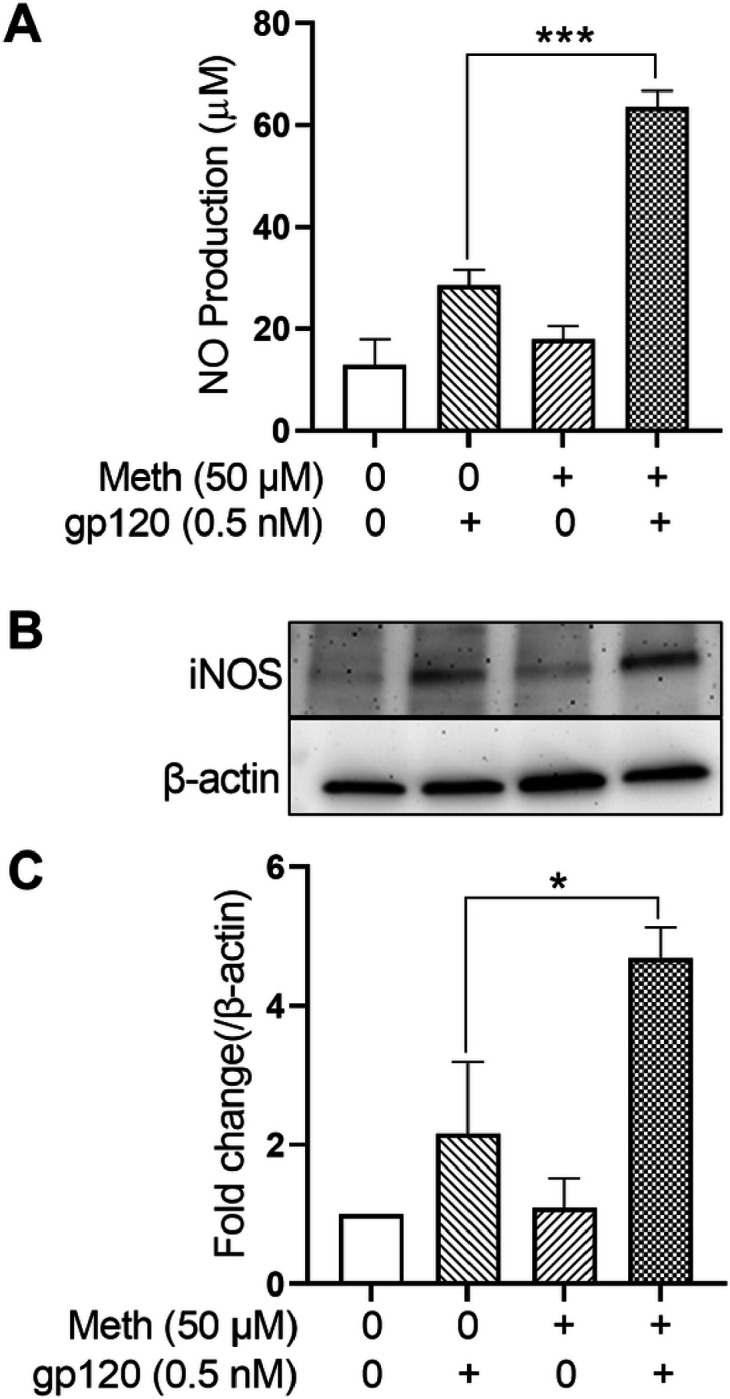
Meth enhancement of iNOS expression and NO production in gp120-primmed microglia. Panel A showed Meth-induced enhancement of NO production in culture supernatants recovered from gp120-primed microglia. Panels B and C exhibited an increase of iNOS expression detected using microglial lysate *p<0.1, ***p<0.001 vs gp120 only treated control.

Studies have shown that activation of microglia causes overproduction of nitric oxide (NO) by inducible nitric oxide synthase (iNOS), resulting in neuroinflammatory processes [48–50]]. To assay the levels of NO production and iNOS expression in gp120-primed microglia treated with Meth, we measured NO production from microglial culture supernatants by ELISA and detected iNOS expression from microglial lysate by western blot. The results showed that the application of Meth to gp120-primed microglial cultures significantly enhanced NO production (Fig. 7A) and iNOS expression (Figs. 7B, 7C).

### Meth enhanced GSDMD-N production in microglial cells primed with gp120

**Figure 8:**
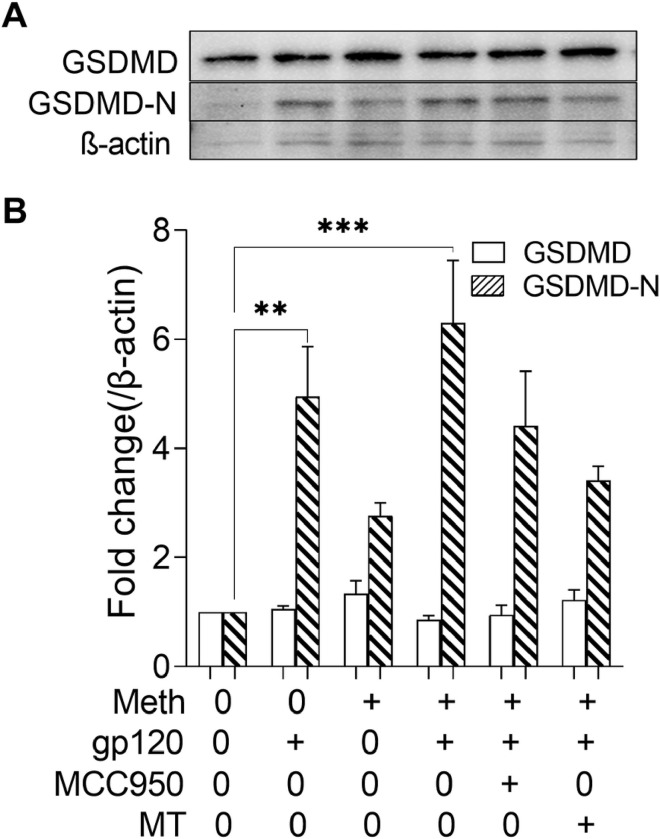
Meth-induced microglial pyroptosis. Panel A was a representative western blot result showing that microglia cells underwent pyroptosis after treatment with gp120, as detected by increased N-terminal GSDMD (GSDMD-N), which was further augmented by Meth treatment. The bar graph in Panel B showed average expression levels of GSDMD and GSDMD-N. Note the augment effect of Meth on gp120-mediated pyroptosis. Data in Panel B represent three independent experiments and each in triplicate. **p<0.01, ***p<0.001 vs untreated control,

Pyroptosis is a form of proinflammatory programmed cell death mediated by caspase-1-cleaved pore-forming protein gasdermin D (GSDMD) [51]. The N-terminal proteolytic fragment of GSDMD (GSDMD-N) is an executor of pyroptosis and is required for IL-1β release [52]. To explore the involvement of NLRP3-dependent pyroptosis in Meth potentiation of gp120-associated pathophysiology, we examined the expression levels of GSDMD-N in microglial cultures. The treatment of microglial cells with gp120 led to cleavage of GSDMD and increased production of GSDMD-N (Fig. 8A). The Meth-enhanced GSDMD-N production in microglial cells primed with gp120 implies an occurrence of inflammatory pyroptosis triggered by Meth. The addition of MCC9500 or Mito-TEMPO to microglial cultures attenuated the Meth-/gp120-associated increase of GSDMD-N production, implying activation of NLRP3/caspase-1 by Meth and gp120.

## Discussion

Although combined antiretroviral therapy (cART) has significantly decreased a spectrum of disease morbidities, including profound dementia, more subtle forms of HIV-1-associated neurocognitive disorders (HAND) remain prevalent [14, 53, 54]. Virus persists in the brain at low levels, often in a latent or restricted manner. Immune activation and neuroinflammation, which are linked to viral proteins and drugs of abuse, continue to play pivotal roles in HAND pathogenesis. Meth abuse exacerbates the HAND seen clinically and the mechanism(s) underlying such an exacerbation remain unclear [23, 55, 56]. To understand how Meth exacerbates HAND we studied the augment effects of Meth on HIV-1 gp120-induced microglial NLRP3 inflammasome activation and resultant proinflammatory cytokine production. Our results revealed that Meth enhanced gp120-stimulated microglial activation and resultant proinflammatory cytokine production via mitochondria-dependent NLRP3 inflammasome activation.

The NLRP3 inflammasome is a critical component of the innate immune system that mediates caspase-1 activation and proinflammatory cytokine production in response to diverse stimuli and multiple biomolecules, including, but not limited to, viral proteins and the drugs of abuse. Typically, two independent signals are required to fully activate the NLRP3 inflammasome [35, 44]. To understand whether Meth potentiates gp120-induced NLRP3 inflammasome activation in microglia, we previously examined the effects of Meth on lipopolysaccharide (LPS, a known priming signal)-induced NLRP3 inflammasome activation in rat microglial cultures. We observed that Meth could potentiate the pre-existing inflammatory stimulation and produce an enhancement in IL-1β maturation and release in an NLRP3 inflammasome-dependent manner. In the present study, we substituted LPS with HIV-1 gp120 to reflect disease conditions in human subjects and investigated the enhancement effects of Meth on HIV-1 gp120-induced rat microglia activation and resultant inflammatory responses, focusing on the involvement of NLRP3 inflammasome. We observed that Meth enhanced NLRP3 inflammasome activation and proinflammatory cytokine production in HIV-1 gp120-primed rat microglial cells. The enhancement effects were attenuated or blocked by the addition of NLRP3 inflammasome inhibitor MCC950 and/or a mitochondria-targeted antioxidant Mito-TEMPO, suggesting involvement of mitochondria-dependent NLRP3 inflammasome in Meth enhancement of gp120-stimulated microglial activation and proinflammatory cytokine production.

Meth is one of the most abused drugs among individuals infected with HIV-1[25]. Ample evidence indicates that Meth abuse exacerbates cognitive deficits and neurodegenerative abnormalities in HIV-1-infected patients and animal models [27, 28, 57, 58]. To explore the impact of Meth on HIV-1 gp120-induced microglial NLRP3 activation, we first examined the individual effects of Meth and gp120 on NLRP3 inflammasome activation in primary rat microglial cultures. Consistent with our previous observations [44] Meth had no significant effects on pro-IL-1β/IL-1β expression and IL-1β release at three different doses (6, 18, 50 μM), indicating that Meth may not work as the first signal for inflammasome activation [44]. However, HIV-1 gp120 produced a significant increase in pro-IL-1β/IL-1β expression and IL-1β release at concentrations of 0.5 nM and higher, suggesting gp120 could cause NLRP3 inflammasome activation. When tested in combination, 50 μM Meth was found to enhance gp120-mediated increase of pro-IL-1β/IL-1β expression and IL-1β release, implying Meth enhancement of gp120-associated NLRP3 inflammasome activation.

HIV-1 gp120 plays an important role in the HAND pathogenesis. It causes immune activation and resultant production of proinflammatory cytokines as well as inflammasome-dependent pyroptosis, in addition to its direct toxic effects on neural cells. The neurotoxic effects of HIV-1gp120 could be potentiated by drugs of abuse, such as Meth. The mechanisms for Meth potentiation of HIV-1gp120-associated neurotoxicity are multifaceted, including, but not limited to, activation of microglial NLRP3 inflammasome. Studies have shown that NLRP3 inflammasome is involved in HIV-1 gp120-associated microglia activation [20, 59] and resultant neuronal injury [32]. In agreement with the abovementioned studies, our results showed that Meth enhanced pro-IL-1β/IL-1β expression, processing and release in HIV-1 gp120-primed microglial cultures which were attenuated or blocked by MCC950, a specific NLRP3 inflammasome inhibitor, demonstrating Meth-induced potentiation of gp120-primed microglial activation via NLRP3 inflammasome signaling in cultured rat microglial cells.

Treatment of microglia with Meth significantly increased the production levels of IL-1β, TNF-α, IL-6 and IL-18 in gp120-primed microglia compared to those treated each alone. The production of these cytokines was significantly reduced by pretreatment of microglia with Mito-TEMPO and MCC950, suggesting an involvement of mitochondria and NLRP3 inflammasome in Meth-associated increase of cytokine production. As mitochondria are an important source of ROS, we detected total ROS activation and observed a significant increase after treatment of microglia with gp120 and Meth, indicating that ROS may play a role in inflammasome activation signaling [60]. In addition to ROS activation, Meth-associated elevation of inducible nitric oxide synthase (iNOS) expression and increase of nitric oxide (NO) production were detected in gp120-primed microglial lysate and culture supernatants, respectively. The increased production of NO could be one of the mechanisms underlying Meth exacerbation of HAND seen clinically.

The Meth potentiation of HIV-1gp120-primed NLRP3 inflammasome activation was supported by experimental results demonstrating the co-localization of NLRP3 inflammasome with its downstream effector protein caspase-1 and formation of NLRP3 puncta as visualized by immunofluorescence microscopy. We observed that the co-localization of NLRP3 and caspase-1 was enhanced upon Meth treatment to the gp120-primed microglial cells, a sign of Meth enhancement of NLRP3/caspase-1 activation. As NLRP3 activation leads to the assembly of NLRP3, ASC and caspase-1 and the formation of NLRP3 inflammasome complex, a micron-sized dense structure known as puncta, Meth was found to increase the numbers of NLRP3 puncta-positive cells, a hallmark of inflammasome activation [61]. Treatment of microglial cells with MCC950 significantly decreased NLRP3/caspase-1 co-localization and puncta formation, indicating an involvement of NLRP3 inflammasome in Meth-associated enhancement of NLRP3/caspase-1 colocalization and puncta formation. In addition to NLRP3 puncta quantification, we measured the levels of NLRP3 expression using immunoblot and found a significant elevation in NLRP3 expression in gp120/Meth-treated microglial cells, which was significantly restored upon pretreatment of cells with MCC950. The detection of an increased colocalization and puncta formation of NLRP3 inflammasome complex inside a cell after Meth treatment to HIV-1gp120-primed microglial cells strongly support Meth enhancement of HIV-1gp120-associated microglial activation.

Canonically, the inflammasome can be activated in response to various upstream signals. As the primary mediator for pro-IL-1β maturation, NLRP3 inflammasome activation is accompanied by the processing of pro-IL-1β, cleavage of caspase-1 and ASC protein aggregation. After sequential stimulation with HIV-1gp120 and Meth, NLRP3 and IL-1β were cleaved to their activated forms as illustrated by their upregulated expression levels and increased proinflammatory cytokine production (e.g., IL-1β, IL-6, IL-18 and TNFα). The increase of proinflammatory cytokine production led to pyroptosis as demonstrated by enhanced expression of GSDMD-N, a central player in executing pyroptosis, the cell death pathway downstream of inflammasome activation [62]. These results were in an agreement with the classical cellular pattern of inflammasome activation, in which principal components redistribute from dispersed to clustered and promote restoration of caspase-1 enzyme activity after the cross-cleavage process [63].

In summary, the present study demonstrated that Meth enhanced microglia activation and proinflammatory responses via NLRP3 inflammasome activation in gp120-promed rat microglial cultures. In addition to the enhancement of gp120-mediated NLRP3 expression, IL-1β processing and release, the augmentation of the co-localization of NLRP3 with caspase-1, and the increase of the numbers of NLRP3 puncta and ROS production, Meth also elevated the levels of iNOS expression and NO production, as well as the level of cleaved gasderminD (GSDMD-N), an executor of pyroptosis, in gp120-primed microglia. The Meth-associated effects were attenuated by MCC950, a NLRP3 inhibitor, or Mito-TEMPO, a mitochondrial superoxide scavenger. These results suggest that Meth potentiated gp120-associated microglial NLRP3 activation and proinflammatory responses via mitochondria-dependent signaling.

## Figures and Tables

**Figure 9:**
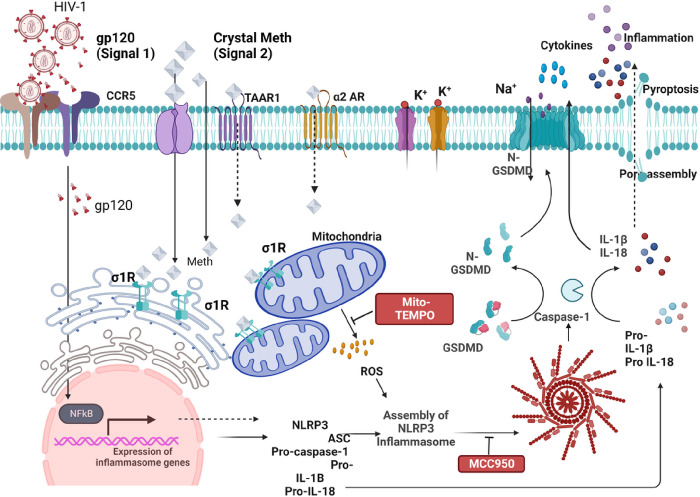
Potential mechanisms for Meth enhancement of NLRP3 inflammasome activation in gp120-primed microglia in a two signal model. Gp120 works as priming (first signal) and induces upregulation of transcription of pro-IL-1βand NLRP3. Meth helps in processing (the second signal) for the NLRP3 inflammasome activation via mitochondria-associated ROS signaling.

## Data Availability

The data supporting this study’s findings are available from the corresponding author upon reasonable request.

## Abbreviations

AIDS: acquired immunodeficiency syndrome
BSA: Bovine serum albumin
cART: combined antiretroviral therapy
CM-H2DCFDA: 5-(and-6)-chloromethyl-2′,7′-dichlorodihydrofluorescein diacetate
DAPI: 4′,6-diamidino-2-phenylindole
DMEM: Dulbecco’s modified Eagle’s medium
ECL: enhanced chemiluminescence
ELISA: enzyme-linked immunosorbent assay
FBS: fetal bovine serum
gp120: HIV-1 envelope glycoprotein protein 120
GSDMD: gasderminD
GSDMD-N: N-terminal proteolytic fragment of GSDMD
HAND: HIV-1-associated neurocognitive disorders
HBSS: Hank’s Balanced Salt Solution
HIV-1: Human immunodeficiency virus type 1
HRP: horseradish peroxidase
IACUC: institutional animal care and use committee
Iba-1: ionized calcium-binding adaptor molecule 1
iNOS: inducible nitric oxide synthase
LPS: Lipopolysaccharide
M-CSF: macrophage colony-stimulating factor
Meth: methamphetamine
NLRP3: nucleotide-binding domain, leucine-rich–containing family, pyrin domain–containing-3
NLR: NOD like receptor
NO: nitric oxide
PVDF: polyvinylidene difluoride
SD: Sprague-Dawley
TBS: tris-buffered saline
